# Enamel biomimetics—fiction or future of dentistry

**DOI:** 10.1038/s41368-018-0038-6

**Published:** 2019-01-05

**Authors:** Mirali Pandya, Thomas G. H. Diekwisch

**Affiliations:** Center for Craniofacial Research and Diagnosis, Texas A&M College of Dentistry, Dallas, TX USA

**Keywords:** Biomimetic synthesis, Molecular medicine

## Abstract

Tooth enamel is a complex mineralized tissue consisting of long and parallel apatite crystals configured into decussating enamel rods. In recent years, multiple approaches have been introduced to generate or regenerate this highly attractive biomaterial characterized by great mechanical strength paired with relative resilience and tissue compatibility. In the present review, we discuss five pathways toward enamel tissue engineering, (i) enamel synthesis using physico-chemical means, (ii) protein matrix-guided enamel crystal growth, (iii) enamel surface remineralization, (iv) cell-based enamel engineering, and (v) biological enamel regeneration based on de novo induction of tooth morphogenesis. So far, physical synthesis approaches using extreme environmental conditions such as pH, heat and pressure have resulted in the formation of enamel-like crystal assemblies. Biochemical methods relying on enamel proteins as templating matrices have aided the growth of elongated calcium phosphate crystals. To illustrate the validity of this biochemical approach we have successfully grown enamel-like apatite crystals organized into decussating enamel rods using an organic enamel protein matrix. Other studies reviewed here have employed amelogenin-derived peptides or self-assembling dendrimers to re-mineralize mineral-depleted white lesions on tooth surfaces. So far, cell-based enamel tissue engineering has been hampered by the limitations of presently existing ameloblast cell lines. Going forward, these limitations may be overcome by new cell culture technologies. Finally, whole-tooth regeneration through reactivation of the signaling pathways triggered during natural enamel development represents a biological avenue toward faithful enamel regeneration. In the present review we have summarized the state of the art in enamel tissue engineering and provided novel insights into future opportunities to regenerate this arguably most fascinating of all dental tissues.

## Biomimetics: Current state and future directions of enamel tissue engineering

Five pathways for tooth enamel engineering hold great promise for developing new technologies, leading to novel biomaterials and biotechnologies to regenerate enamel tissue. Tooth enamel is a unique tissue-specific biomaterial with exceptional structural and mechanical properties. In recent years, many approaches have been adopted to generate or regenerate this complex tissue; Mirali Pandya and Thomas Diekwisch of Texas A&M College of Dentistry, USA conducted a review of the current state and future directions of enamel tissue engineering. In their review, the authors focused on five pathways for enamel tissue engineering: (1) physical synthesis of enamel; (2) biochemical enamel engineering; (3) in situ enamel engineering; (4) cell-based enamel engineering; and (5) whole tooth regeneration. The authors conclude that those five approaches will help identify the biological mechanisms that lead to the generation of tooth enamel.

## Tooth enamel—an impossible material to regenerate?

**Fig. 1:**
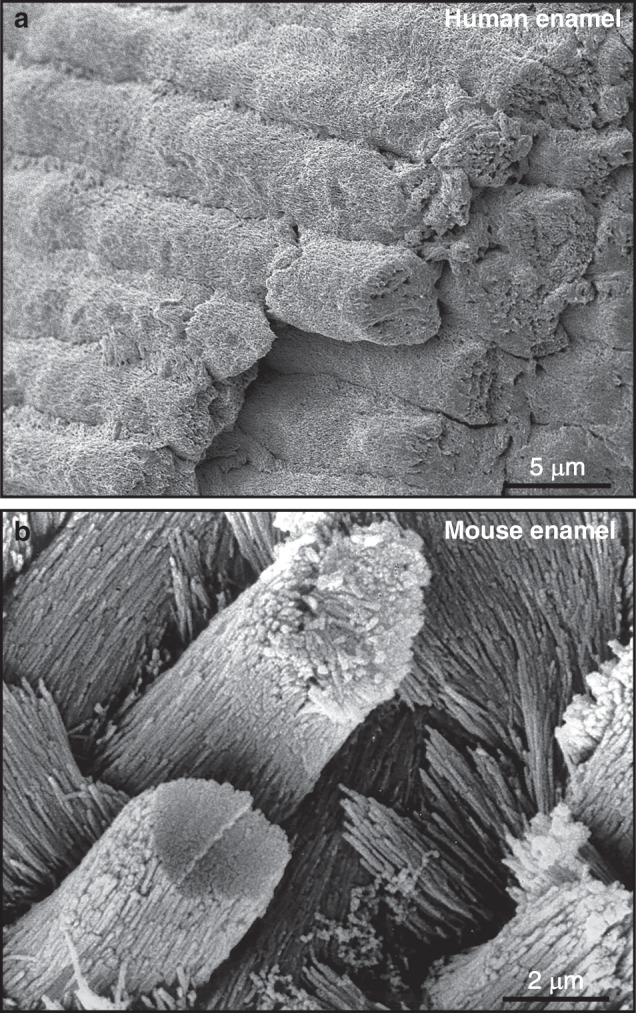
Scanning electron micrographs of mammalian enamel topography. **a** Human enamel. Note the densely packed apatite crystal network organized into cylindrical enamel prisms (rods). **b** Mouse enamel. Individual subunits within each prism are clearly delineated

Tooth enamel is a highly unique tissue-specific biomaterial characterized by exceptional structural and mechanical properties as well as esthetic beauty.^1–4^ The unique physico-chemical properties of enamel are due to its high content in hydroxyapatite, the parallel arrangement of individual elongated apatite crystals into enamel prisms, and the interwoven alignment of perpendicular prisms in a picket-fence resembling three-dimensional order (Fig. 1). Together, these characteristics result in a biomaterial of great hardness and physical resilience. Due to its toughness and relative fracture resistance, enamel-like biomaterials hold great promise as structural components for future biomedical and engineering applications, including tooth enamel repair, orthopedic defect restoration, and as functional components of insulators, brakes, and exhaust pollutant filters.^5–9^

As desirable as the regeneration or fabrication of tooth enamel may seem, de novo enamel tissue engineering and its potential future clinical implementation remain a daunting task.^10–13^ In biological organisms, enamel is manufactured only once prior to tooth eruption, and the capacity to form new enamel in each individual tooth organ is lost forever, once the tooth is fully erupted.^14,15^ The high ion concentrations and dramatic pH changes involved in initial amelogenesis pose a formidable hurdle in cell-based approaches toward tooth enamel regeneration.^16–18^ And even though the synthesis of hydroxyapatite blocks may appear straight-forward from a manufacturing perspective, the faithful fabrication of true enamel with its parallel-aligned filigree apatite crystals and decussating prism bundles has rarely been accomplished so far.^19–23^

The cells at the core of nature’s ability to manufacture tooth enamel are called ameloblasts. Ameloblasts are highly specialized epithelial cells originally derived from the enamel organ. After differentiating from inner enamel organ cells and thereafter pre-ameloblasts, ameloblasts turn into highly polarized and elongated prismatic cells with a pronounced endoplasmic reticulum and Golgi apparatus to synthesize and secrete amelogenin and other enamel proteins and transport calcium and phosphate ions into the enamel matrix. Once a sufficient amount of enamel matrix has been synthesized, ameloblasts function to resorb large quantities of water and degraded enamel matrix proteins during the resorptive stage of enamel formation. While it appears logical to culture ameloblasts for the in vitro manufacture of tooth enamel, ameloblast culture approaches have encountered numerous difficulties, perhaps due to the highly differentiated status of these secretory cells or due to the lack of a suitable tissue context and/or related physical cues. In comparison, ameloblast precursor cells and stratum intermedium ameloblast progenitor cells have been relatively easier to maintain in vitro, but so far have not demonstrated any evidence of enamel matrix secretion in culture. In contrast, maintenance of postsecretory ameloblasts in vitro has remained challenging because of their reduced proliferative capability. Finally, cells from the papillary layer and junctional epithelium would require extensive reprogramming for tissue engineering purposes because of their physiological inability to secrete amelogenin and/or transport mineral. As a result, cellular approaches for enamel regeneration require novel strategies to reach a level of proficiency that is customary in other cellular regeneration models.

Two recent conferences related to tooth enamel (Enamel IX and the “Encouraging Novel Amelogenesis Models and Ex vivo cell Lines (ENAMEL) Development workshop”) have outlined some of the knowledge gaps that have so far prevented the enamel field from being able to address the challenges in enamel regeneration and engineering, including its cell-free nature, its high mineral content, and its unique structural organization.^24,25^ However, during the recent decade, several laboratories have developed innovative approaches to either synthesize or engineer enamel-like tissues or to culture enamel-secreting cells and tissues and thus mimic aspects of enamel development. Here we have summarized and reviewed current approaches as a guide for future experimental strategies toward enamel biomimetics and proposed novel concepts that will hopefully benefit future efforts toward enamel regeneration and engineering.^24–27^

## Physical synthesis approach

In nature, the conversion of inorganic calcium phosphates into crystalline apatites requires extreme conditions such high temperature, high pressure, or unusual pH. Any synthetic process seeking to manufacture hydroxyapatite as the principal component of biosynthetic tooth enamel would need to mimic the biological conditions required for apatite biomineralization and generate an environment that resembles some of the extreme environments that occur during physiological hydroxyapatite crystallization. Therefore, physical approaches toward enamel synthesis rely on extreme conditions in terms of temperature, pressure, or isoelectric point, or a combination thereof.

The first synthetic generation of apatite nanorods was based on an aqueous solution of hydroxyapatite titrated to pH 2 in conjunction with surfactant docusate sodium salt as a colloidal suspension solution.^28^ Adjusting this solution to only slightly acidic conditions (pH 5.8) resulted in the precipitation of 200–400 nm long apatite crystals with a Ca/P (Calcium/Phosphate) ratio of 1.6, fairly close to atomic Ca/P ratio of hydroxyapatite at 1.67.^28^ This study represented the first successful approach toward the synthetic generation of parallel-aligned and elongated enamel-like apatite crystals.^28^ To generate apatite nanorods that more closely matched the size of natural enamel crystals, the hydroxyapatite solution from the previous study was replaced with a fluorapatite solution, and the atmospheric conditions were altered to include intense hydrothermal pressure by autoclaving the crystallization solution for about 10 h on an iron plate substrate. The fluorapatite crystals generated using this hydrothermal pressure approach measured ~5–10 µm in cross section, similar to the size of human enamel crystals.^29^

A third synthetic approach was devised to avoid some of the extreme conditions employed as part of the previous two approaches, namely high pressure, high acidity, and the use of toxic nucleation or emulsification conditions. Instead, this third approach relied on high temperature (150–200 °C for up to 72 h), a sodium bicarbonate buffer to regulate the pH during crystal formation, and a crystallization solution consisting of calcium nitrate tetrahydrate, di-sodium hydrogen phosphate, sodium bicarbonate, and octacalcium phosphate at a pH of 6.6. The apatite rods that resulted from these experiments were substantially smaller than human enamel crystals, measuring between 200 nm and 500 nm in length, 100 nm and 200 nm in width, and a stoichiometric calcium/phosphate ratio up to 1.67.^30^

Recently, a three-step synthetic process was conceived to mimic key aspects of initial enamel formation, including (i) conjugation of carboxymethyl chitosan (CMC) with alendronate (ALN) to stabilize amorphous calcium phosphate (ACP) and form CMC/ACP nanoparticles, (ii) application of sodium hypochlorite (NaClO) to degrade the CMC-ALN matrix generated in step (i), and (iii) use of 10 nmol•L^−1^ glycine (Gly) to guide HAP/ACP (hydroxyapatite/ amorphous calcium phosphate) nanoparticles to organize into well-ordered rod-like apatite crystals.^31^ This process is based on a polysaccharide/bisphosphonate matrix (chitosan/alendronate) and mimics key steps of initial amelogenesis, including (i) formation of a Ca/P-rich amelogenin protein matrix, (ii) enzymatic degradation and continued crystal growth, and (iii) crystal elongation as facilitated by elongated amelogenin fragments.^32^ In their paper, the authors suggested that a combination of non-classical crystallization mechanisms, development of synthetic amelogenin analogues, and imitating remaining biomineralization steps would hold great promise for future approaches to improve the repair of enamel defects.^31^

Together, the four studies summarized above have made substantial progress toward the goal of synthesizing enamel-like structures in vitro. However, current approaches still rely heavily on synthetic conditions such as high temperature, pressure, or extreme pH, or employ toxic chemicals such as surfactant, bisphosphonate, or sodium hypochlorite, preventing such synthetic approaches from immediate application in the oral cavity. Moreover, soft intermediate materials as they would be generated during the three-step chitosan/glycine-based synthesis would not withstand the masticatory pressures on occlusal surfaces during mastication. Nevertheless, the simple feat of synthesizing enamel-like apatite materials en block will avail a future generation of dental practitioners with highly biomimetic materials that may replace parts of the enamel layer or the entire enamel layer when used in combination with digital grinding and milling technologies.

## Biochemical enamel engineering

Tissue engineering has often been described as a means to copy developmental biology for regenerative purposes. Mimicking natural tooth enamel formation for tissue engineering purposes would involve the manufacture of an amelogenin-rich protein matrix and enriching this matrix with calcium phosphate ions. Following initial apatite crystal formation, this cocktail would then be subjected to enzymatic processing using enamel matrix proteases such as matrix metalloprotease 20 (MMP20) and kallikrein 4 (KLK4), presumably resulting in initial *c*-axis crystal elongation and thereafter lateral crystal growth in an *a*- and *b*-axis direction. As it turns out, enamel development is exponentially more complex in nature, and the simplified approach mentioned above has not yet been successful in the laboratory. Challenges encountered when using a simple developmental approach toward enamel tissue engineering include mimicking the coordinated movement of ameloblast cells as the formative units associated with the secretion of each individual prism, adjusting the pH value of the mineralization solution in a dynamic fashion as it occurs in vivo, counteracting the inhibitory effects of the amelogenin protein on crystal growth and selectively applying individual amelogenin fragments in a biomimetic fashion to control apatite crystal growth. The multidimensional symphony of events that eventually results in natural enamel complexity is still too little understood to propose a cookbook formula for the generation of enamel in a test-tube.

Developmental biology has informed us that the initial enamel matrix consists to 60–70% of water, 20–30% of proteins, and 15–20% of mineral ions.^33,34^ Three unique matrix proteins have been associated with the developing enamel matrix, amelogenin, ameloblastin, and enamelin, which have therefore been coined enamel proteins.^35^ Among these, amelogenin is by far the most abundant protein component in the developing enamel layer, contributing to more than 90% of its overall volume.^36–38^ Other proteins that play a substantial role during amelogenesis include enamel matrix proteases such as matrix metalloprotease 20 (MMP20) and kallikrein 4 (KLK4) that facilitate the posttranslational processing of enamel matrix proteins.^39–42^ Together, the enamel proteins are thought to contribute to the three major functions of the developing enamel matrix, i.e., (i) enamel hydroxyapatite crystal nucleation, (ii) enamel apatite *c*-axis crystal growth, and (iii) the spacing between individual apatite crystals during crystal nucleation and growth.^32,43–49^ Only upon completion of mineralization, enamel proteins and water are resorbed from the developing enamel layer, resulting in a 1% organic matter content in the mature enamel, while the remaining 99% volume contains inorganic material, mostly apatite.^47^

**Fig. 2:**
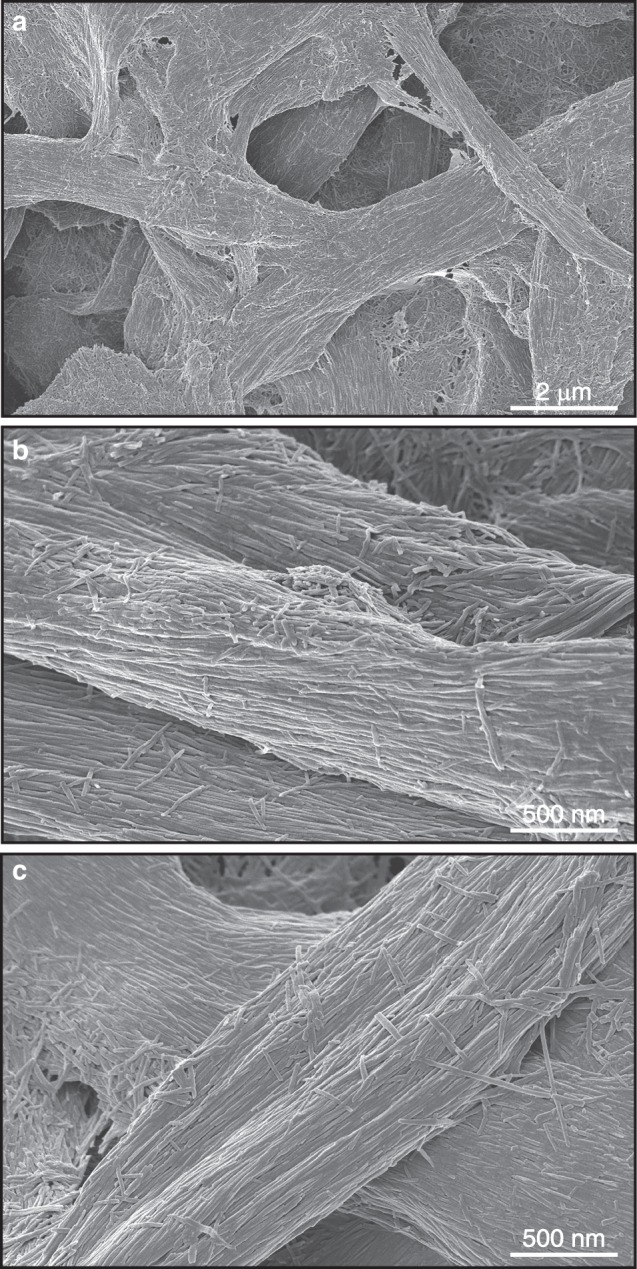
Scanning electron micrographs of engineered enamel. In this study apatite was grown within a decellularized enamel protein matrix, resulting in decussating enamel prisms containing distinct and separated individual enamel crystals. **a** is an overview scanning electron micrograph of the engineered enamel apatite, **b** demonstrates parallel bundles of enamel crystals, and **c** illustrates newly generated decussating enamel rods

Based on the interaction between majority components, combinations of enamel proteins and calcium phosphate growth solutions would be a logical first step toward the biological synthesis of tooth enamel. In support of this approach, we have been able to grow elongated and parallel apatite crystals within decussating enamel prisms using an enamel protein matrix (Fig. 2). Earlier studies using metastable octacalcium phosphate growth solutions in combination with a 10% (w/v) amelogenin gel have yielded elongated octacalcium phosphate crystals of limited length and thickness,^50–52^ or apatite crystals after addition of fluoride.^53,54^ It has also been demonstrated that a cooperation between amelogenin and another enamel protein, enamelin, resulted in the stabilization of the amorphous calcium phosphate precursor phase and an increase in the length to width ratio of resulting octacalcium phosphate crystals, likely due to a co-assembly between amelogenin and enamelin.^55,56^

While combinations of octacalcium phosphate crystal growth solutions and amelogenins succeeded in the growth of calcium-rich parallel-oriented crystal bundles, this approach did not result in enamel-like biomaterials in terms of size, hardness, and structure. Yet, these studies re-affirmed the basic concept that enamel proteins promote enamel-like apatite crystal growth in vitro. To further improve on the concept of enamel protein-guided crystal growth, a number of studies have focused on the alternative splicing and enzymatic cleavage processes that are a prominent feature of enamel development. These studies indicate that the presentation of amelogenin fragments and other cleavage products to the nucleating calcium phosphate crystals and elongating apatite surfaces is of functional significance for proper enamel crystal growth.^32,57^ So far, studies have focused on the hydrophilic amelogenin C-terminus, the LRAP (leucine-rich amelogenin peptide) splicing product, and the use of the MMP20 metalloproteinase to promote amelogenin processing. While each of these strategies further improved on aspects of enamel formation, future studies have yet to capture the cascade of events and the interactions between individual fragment functions to mimic the multidimensional complexity of mammalian enamel crystal growth.

Recent studies have emphasized the close proximity of the amelogenin hydrophilic C-terminus to the growing enamel crystal surface and its role in promoting crystal growth.^32,58^ The functional importance of the amelogenin C-terminus prompted a group of investigators to synthesize oligopeptides that structurally resembled amelogenin by combining amelogenin C-terminal hydrophilicity with a derivative of stearic acid (C_18_H_35_COOH) as the hydrophobic end of an amphiphile.^59^ These amphiphiles self-assembled, formed 12 nm wide nanofibers, and provided a template for amorphous calcium phosphate (ACP) growth in a metastable calcium phosphate solution.

Another approach focused on the leucine-rich amelogenin peptide, a 59-residue amelogenin fragment created during amelogenin alternative splicing.^60,61^ In this study, two porcine LRAP modifications were synthesized, phosphorylated ( + P) LRAP and non-phosphorylated (−P) LRAP, and added to a calcium and phosphate-rich solution. These peptides displayed a tendency to form spherical nanoparticles of 10–12 nm diameter and assembled in a linear chain-like pattern. The self-assembled peptides formed spherical ACP particles and triggered ACP to hydroxyapatite (HA) phase transformation accompanied by a drop in pH.^62^

A third approach to determine the effect of amelogenin fragmentation products on enamel crystal growth encompassed the addition of the MMP20 metalloproteinase to an amelogenin matrix combined with a chitosan gel and a calcium phosphate crystal growth solution.^63,64^ This scenario somewhat resembled in vivo conditions as previous studies had indicated that MMP20 null mice suffered from retention of organic matrix and low crystallinity compared to wildtype (WT) mice.^58,64,65^ Here, the addition of MMP20 promoted a break-down of amelogenin after initial crystal formation, resulting in improved biomechanical qualities of the newly generated apatite crystals.^64^

So far, studies using a biochemical approach have only mimicked individual aspects of apatite and calcium phosphate crystal growth. Additional improvements would result in biomaterials that more closely resemble natural enamel which then could be applied to the patient’s mouth.

## In situ enamel engineering (enamel surface remineralization)

Once teeth are erupted, the cells and tissues involved in enamel formation, i.e., the ameloblasts and the enamel organ, are no longer present on the tooth surface. The lack of natural means for enamel regeneration has created an opportunity for restorative dentistry and for the replacement of enamel tissues with synthetic substitutes such as amalgams, gold, porcelain, and polymer composites. The advent of biomimetics has led to an exploitation of natural mechanisms that either alter tooth enamel apatite mechanical and chemical surface properties or to grow layers of enamel-like apatite material onto the surface of already existing tooth enamel, using enamel protein-like substrates together with apatite growth solutions.

The benefits of fluorides for dental health and resistance of tooth enamel against tooth decay are known since the groundbreaking studies of Frederick McKay and Greene Vardiman Black on the effects of fluoride on mottled and cavity-free teeth, and the drinking water fluoridation studies in Grand Rapids, Michigan, by H. Trendley Dean.^66^ Fluoride affects enamel properties through the exchange of the hydroxyl group (-OH) in the hydroxyapatite Ca_5_(PO_4_)_3_OH with a fluoride ion to form either fluorapatite or fluor-hydroxyapatites.^67,68^ The fluoride-substituted apatite is harder and less soluble when compared to fluoride-free apatite and hence more resistant to acidic environments, which explains the use of fluoride in toothpastes and varnish gels as a means to prevent caries-related demineralization. One of the downsides of excess fluoride levels is the development of brown and mottled teeth (fluorosis).^69^ Future technologies may result in the development of alternatives to fluoride with less side effects that would nevertheless improve the mechanical properties of enamel and its caries resistance.

Other approaches have focused on enamel remineralization strategies through toothpastes. For example, combinations of full-length recombinant pig amelogenin rP172 and fluoride resulted in the formation of a fluoridated calcium phosphate coat with needle-like crystal bundles on an etched enamel surface.^70^ An in vivo study based on a gelatin gel in conjunction with calcium and fluoride ions to treat enamel surface defects demonstrated formation of a smooth enamel-like layer, but little is known about the long-term success rates, hardness or structural integrity of the newly formed enamel layer.^71^

A third biomimetic approach toward enamel surface remineralization has focused on the natural ability of tooth enamel proteins to nucleate and guide the growth of enamel apatite crystals. Earlier studies have demonstrated that full-length tooth enamel proteins such as amelogenins and enamelins inhibit apatite crystal growth in vitro.^72^ Thus, it has long been assumed that alternative splicing and posttranslational cleavage of enamel proteins are essential for the ability of the enamel protein matrix to promote enamel crystal growth.^32^ The following individual amelogenin fragments have been correlated with unique functions related to enamel crystal growth: (i) the hydrophobic amelogenin N-terminus including its TRAP (tyrosine-rich amelogenin peptide) domain has been associated with matrix self-assembly,^73^ (ii) the central polyproline repeat region has been speculated to control crystal spacing,^74^ and (iii) the hydrophilic C-terminus has been demonstrated to facilitate amelogenin protein solubility and its adhesion to the crystal surface.^32,75^ In addition to these relationships between amelogenin functional domains and crystal growth, there has also been interest in two prominent polypeptides in the developing enamel matrix, the tyrosine-rich amelogenin peptide containing the amino-terminal 44–45 amino acids of the full-length amelogenin (TRAP), and the leucine-rich amelogenin peptide (LRAP), which includes both the N-terminal 33 amino acids and the C-terminal 26 amino acids.^60^ The importance of LRAP for enamel crystal formation has been confirmed in mouse models in which LRAP successfully rescued the enamel null mouse phenotype.^76^ Supportive of a putative role of LRAP as an amelogenin isoform capable of promoting both enamel crystal nucleation and growth, LRAP improved the remineralization of enamel surface lesions on extracted teeth in vitro more effectively than full-length amelogenin.^77–81^ A combination of LRAP with a chitosan-based hydrogel resulted in the formation of a dense enamel-like apatite layer on the surface defect.^80^ From a mechanistic perspective, LRAP might combine some of the self-assembly properties of the amelogenin N-terminus with the hydrophilic properties of the C-terminus to promote *c*-axis elongation of enamel crystals.^82^ Another tooth enamel matrix product, the enamel matrix derivate Emdogain® (EMD), has become one of the first successful commercial applications of protein extracts deliberately applied in tissue regeneration procedures. EMD is composed of enamel matrix proteins extracted from porcine teeth and has been widely used for periodontal tissue regeneration in clinical settings.^83–86^ Revealing its potential for enamel tissue engineering, EMD formed enamel prism-like structures in combination with a chloride-based agarose hydrogel. The Ca/P ratio of the resulting mineralized structures was 1.69, close to that of natural enamel apatite crystals.^87^

In addition to natural proteins, synthetic self-assembling peptides have been developed as agents to promote the remineralization of the white lesions associated with initial caries in humans. So far, three peptides are known to promote early caries/white lesion remineralization (i) an amelogenin repeat-based peptide consisting of 22-residues in five tandem amelogenin polyproline repeats (GLn-Pro-X) and a 7 residue hydrophilic tail,^88^ (ii) a triplet repeat of asparagine-serine-serine or 3NSS based on the aspartic-serine-serine repeat sequences in dentin phosphoprotein,^89^ and (iii) a β-sheet forming self-assembling peptide termed P_11_-4^90^ that has proven successful in clinical studies.^91–94^ In addition, a fourth-generation polyamidoamine dendrimer (PAMAM-PO_3_H_2_) with remarkable similarity to amelogenin in terms of its self-assembling potential and an ability to induce crystal remineralization in *c*-axis direction in vivo has been generated.^95^

In summary, recent strategies to achieve enamel tissue regeneration through surface remineralization have shown promising data, suggesting that further studies are likely to improve the integration of the newly synthesized apatite layer with the already existing enamel and enhance the thickness and mechanical properties of the regenerated enamel.

## Enamel organ cell culture and enamel tissue engineering

Classic tissue engineering approaches rely on the cooperation between tissue-specific cell populations, suitable scaffolds, and inductive factors to initiate a cascade of events that leads to the de novo formation of a tissue or organ once lost to trauma or disease.^96,97^ While traditional tissue engineering approaches have been fairly successful in several organs,^98,99^ enamel is unique in that the enamel-forming cells, the ameloblasts, as well as the enamel organ stem cells are lost at the time of tooth eruption.^100^ Ameloblasts are highly specialized epithelial cells as their reverse polarization positions the nucleus at the basal end and the Golgi apparatus at the apical end.^101,102^ Adding to the difficulties of enamel tissue engineering, ameloblast cell culture models have proven to be challenging.^25,100^ Moreover, enamel crystal nucleation and elongation requires a complex array of posttranslational protein modifications,^32^ while the organization of enamel crystals into prisms and the formation of decussating prism patterns depends on highly coordinated ameloblast movements governed by mechanisms still to be discovered.^103^ As a result, there are no reports of successful cell-based in vivo enamel tissue engineering to date.

It is widely accepted that the generation of a potent and viable ameloblast cell line would be a major step toward successful enamel tissue engineering.^25^ So far, five different ameloblast-like cell lines have been reported in the literature, in addition to various protocols for the culture of primary ameloblast-like cells. Successful enamel organ primary cell culture appears to rely on the presence of a supporting feeder cell layer consisting of NIH 3T3 mouse fibroblast cells and a suitable three-dimensional (3D) environment such as a collagen sponge scaffold^104–106^ to either provide underlying support or to substitute for some of the epithelial–mesenchymal interactions that take place during early tooth morphogenesis.^107^ Primary enamel organ cells grown on feeder cell layers have been reported to express amelogenin, ameloblastin, MMP20, kallikrein 4, and other enamel-related proteins.^106^ In addition to enamel organ primary cell culture protocols, three of the five known enamel organ-derived cell lines have been reported to mimic ameloblast-like qualities, the mouse ameloblast-lineage cell line (ALC), the rat dental epithelial cell line (HAT-7), and the mouse LS8 cell line. Two other cell lines have been established but not been used frequently, the porcine PABSo-E cell line^108^ and the rat SF2-24 cell line.^109^

The ALC was initially established from C57BL/6 J mice as a spontaneously immortalized cell line.^110^ This cell line grows on collagen I coated cell culture plates and requires addition of epidermal growth factor (EGF) to the medium. The ability of ALC cells to express amelogenin and tuftelin is suggestive of their similarity to ameloblasts.^110^ Recently, this cell line was used to study the role of phosphate membrane transporters during amelogenesis.^111^

The HAT-7 cell line was generated from the apical end of the incisors of 6 days old rats and cultured in conjunction with BCPb8 cementum progenitor cells and a collagen sponge to create a construct that mimics the epithelial–mesenchymal interactions during early tooth morphogenesis.^112^ HAT-7 cells expressed increased levels of amelogenin and ameloblastin, illustrating the benefits of the extracellular matrix for in vitro amelogenesis.^106^ Based on their polarized nature, HAT-7 cells have been used to trace ion transport in ameloblasts during enamel formation.^113,114^ In another study, HAT-7 cells have been employed to study the role of the glycosphinolipid Gb4 in its role to promote dental epithelial cells to become ameloblasts.^115^ Indicative of their enamel organ lineage, HAT-7 cells differentiated into stratum intermedium-like cells when treated with recombinant human LRAP.^116^

The LS8 cell line is the oldest and most widely used cell line for the study of various aspects of amelogenesis, including signaling pathways and cytokine dynamics.^117,118^ This cell line was originally established almost three decades ago by introducing a Simian virus 40 (SV40) plasmid construct into enamel organ epithelium cells.^119^ When compared to ALC cells, LS8 cells exhibited higher levels of *Amelx, Ambn*, and *Enam* and *Mmp20* mRNAs, while ALC cells expressed higher levels of *Odam*, and *Klk4* mRNAs.^120^ However, none of these cell lines have been reported to form enamel-like structures in vitro, most likely due to their undifferentiated stage.^117,121^

**Fig. 3:**
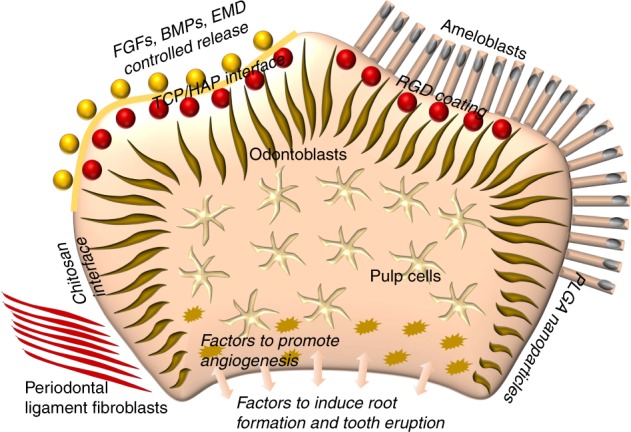
Smart interphase design for enamel tissue engineering within bioreactor environments. In this sketch, suitable growth factor/cell/matrix combinations are proposed to facilitate the differentiation of odontogenic tissues, including enamel, alongside predesigned materials/scaffold interfaces

While these cell lines and primary cells address various aspects of amelogenesis and ameloblast function, none of them resembles the multifunctional enamel organ derivatives that are responsible for enamel formation during tooth development. This may be due to the origin, developmental stage and level of differentiation of the cells chosen so far or caused by the lack of environmental context, both in terms of extracellular matrix and neighboring tissue environment. Immortalized ameloblasts would inherently lack the neighboring stem cell layer of the stratum intermedium,^100^ while immortalized stratum intermedium and stellate reticulum-based lines lack the level of differentiation necessary for the secretion of enamel proteins. Moreover, immortalization is associated with a substantial degree of dedifferentiation,^122,123^ prohibiting ameloblasts from maintaining the level of differentiation necessary for the secretion of amelogenins and other enamel proteins. As a result, other sources for ameloblast stem cells have been explored, including cervical loop stem cells, epithelial cell rest of Malassez (ERM), induced pluripotent stem cells (iPSC), and keratinocytes.^124–129^ Combinations of odontogenic stem cells together with suitable scaffolds and tailored growth factor combinations are likely going to result in epithelial–mesenchymal interfaces suitable for long-term culture (Fig. 3) and benefit the maintenance of stable ameloblast-like cell lines for enamel tissue engineering purposes.

## Whole-tooth regeneration approach

**Fig. 4:**
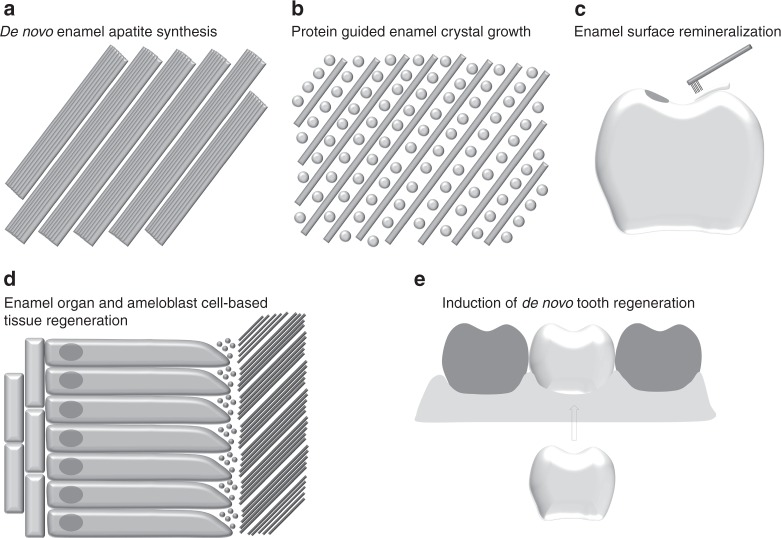
Summary of the five enamel engineering strategies discussed in the present article, including **a** de novo enamel synthesis, **b** protein matrix-guided enamel crystal growth, **c** surface remineralization of white lesions and fluoride applications, **d** enamel organ and ameloblast cell-based tissue regeneration, and **e** induction of de novo tooth regeneration

The previous four chapters have each focused on a singular approach toward enamel tissue engineering, either through physical synthesis, or through biochemical templating and control of enamel crystal growth, or via surface remineralization, or by instructing sheets of ameloblast-like cells to secrete enamel proteins and aid calcium phosphate ions to convert into apatite crystals (Fig. 4). While each of these individual approaches has its own conceptual advantages, a synthesis of their strengths in form of a regenerative medicine approach would have the greatest likelihood to faithfully regenerate “true” enamel with its highly directed and parallel apatite crystals and decussating bundles of enamel rods. As discussed earlier, the immediate drawback of such an approach is the lack of tissues with regenerative capacity to form enamel immediately after tooth eruption. As a result, the success of regenerative approaches toward de novo enamel fabrication is intimately linked to the ability to successfully regenerate whole-tooth organs.

Regenerating whole-tooth organs has long been considered the penultimate dream of dental regenerative medicine. During initial tooth development, epithelial and mesenchymal tissues interact, form bud-stage tooth organs, and continue to develop and differentiate into odontogenic tissues, including ameloblasts, odontoblasts, and cervical loop cells.^107^ From a conceptual point of view, mimicking those signaling cascades to induce de novo tooth formation at any less differentiated epithelial–mesenchymal interface appears to be a logical next step. However, the progression of bud-stage epithelial–mesenchymal interfaces into fully differentiated teeth has proven to require unique structural and inductive environments.^130^ One such example for successful whole-tooth restoration has recently been accomplished by bioengineered tooth-germ transplantation into a donor model.^131^ However, this approach relies on the utilization of reconstituted canine tooth-germ cells.^131^

The ability of cap stage tooth organs to form fully differentiated dentin and enamel mineralized tissues when explanted onto Trowell organ culture dishes has been known for decades.^21,132^ This Trowell organ culture model represents a viable model to grow thin layers of fully developed prismatic enamel in vitro.^22^ Cap stage tooth organs may also be transplanted into the kidney capsule or into the anterior chamber of the eye, yielding further advanced stages of enamel deposition.^133–135^ With rapid advances in three-dimensional cell culture technology, application of ameloblast-specific factors to further the growth and differentiation of enamel-like tissues will harness the natural ability of the enamel organ to manufacture prismatic enamel with mechanical properties similar to human enamel. In addition, computer aided design/computer aided manufacture preparations of biomimetic enamel grown in bioreactors may evolve into enamel repair materials for caries lesions.

From a clinical perspective, it is not clear whether synthetic or regenerated enamel will ever become a mainstream biotechnology product used in future dental offices. Rather, enamel-like biomaterials are likely to find use in many other biomedical or engineering applications because of their enormous strength, resilience, and biocompatibility. With the advancement of regenerative procedures, it is equally imaginable that whole-tooth regeneration approaches will 1 day be applied to address the need to repair or replace diseased or lost dental tissues. A number of genes and pathways have been associated with the regulation of tooth number and tooth development, including Sprouty/fibroblast growth factor (FGF) signaling,^136^ the Ectodysplasin A (*EDA*) gene and its Dickkopf 4 (DKK4)/ Wingless (Wnt) targets,^137,138^ other members of the Wnt/β-catenin family,^139^ as well as imbalances in the WNT/Sonic hedgehog (SHH) homeostasis.^136,140–142^ Gene products associated with supernumerary teeth may be applied in combination with suitable delivery systems to induce de novo tooth formation in areas outside of the dental arch, and these supernumerary teeth may then be autotransplanted to substitute lost or diseased teeth.^143–145^

## Future Directions

To this date, enamel tissue engineering remains a unique biotechnology challenge. Progress in enamel bioengineering is limited partially due to the high level of specialization and interconnectivity of the cells involved in enamel deposition, and also because of the highly evolved materials properties of biological enamel. De novo synthesis approaches will benefit from further insights into the physico-chemical conditions necessary for faithful apatite crystal synthesis and robotic layer deposition to mimic the prismatic organization of biological enamel. Biochemistry-based approaches toward enamel regeneration may employ novel 3D-bioprinting technologies and combine orderly matrix/mineral deposition together with enzymatic matrix degradation steps. Surface biomineralization approaches will improve with advances in peptide design and dendrimer technology. Innovations in bioreactor technology are destined to decipher the optimum environments and conditions necessary for enamel organ and ameloblast cell cultures. Finally, progress in whole organ engineering techniques in combination with novel insights into tooth induction and maintenance signaling cascades will provide sound approaches for future de novo tooth organogenesis (Figs. 3, 4). Together, the five pathways for tooth enamel engineering described here are destined to result in a host of new technologies that not only will generate new biomaterials and biotechnologies for regenerative medicine but also further uncover the unique biological mechanisms that contribute toward the generation of this most unique biological tissue: tooth enamel.
